# Retrospective evaluation of cystic decompression and marsupialisation procedures in odontogenic cysts

**DOI:** 10.6026/973206300211875

**Published:** 2025-07-31

**Authors:** Shreepriya Miskin, Ankush Bhandari, Mahesh Eknath Pund, Rakesh Chanda, Vinay Rao, Sameer Gupta

**Affiliations:** 1 Department of Oral and Maxillofacial Surgery, SJM Dental College and Hospital, Chitradurga, Karnataka, India; 2 Department of Oral and Maxillofacial Surgery, School of Dental Sciences, KVV, Karad, Maharashtra, India; 3 Department of Oral and Maxillofacial Surgeon, GDCH, Chhatrapati Sambhajinagar, Maharashtra, India; 4 BDS, MNR Dental College and Hospital, Telangana, India; 5 Department of Conservative Dentistry, Endodontics and Aesthetic Dentistry, AMC Dental College, Ahmedabad, Gujarat, India; 6 Department of Oral & Maxillofacial Surgery, Inderprastha Dental College and Hospital, Sahibabad, Ghaziabad, Uttar Pradesh, India

**Keywords:** Cystic decompression, marsupialisation, odontogenic cysts

## Abstract

The most common jaw lesions, the odontogenic ones, are treated with less radical surgeries such as decompression as well as marsupialization, which have now been shown to be a substitute. To compare the clinical efficacy of the two methods, the results of 124 patients under treatment between 2018 and 2022 were analyzed in accordance with a retrospective study. Decompression was found to reduce the cyst size (78.4 % vs. 71.6 %) and also minimize the treatment period (8.3 vs. 11.7 months) as compared to marsupialization. The improvement of bone density was close in both groups, the complication rate was low and recurrences were minimal. These results advocate the use of decompression as a more productive conservative treatment, in big cysts and young patients.

## Background:

The Odontogenic cysts are the most common benign bone lesions of the maxillofacial area and originate from the remains of the odontogenic epithelium and are localized only in the tooth-bearing parts of the jaws [1]. Various subtypes of these pathological entities include radicular cysts, dentigerous cysts and keratocystic odontogenic tumors that have different clinical and histopathological features [2]. Classical surgical treatment of the odontogenic cysts has mostly been enucleation, which may be successful with the possibility of complications, including the risk of damaging the surrounding essential structures and destruction, in the treatment of large-sized lesions [3]. Traditional radical surgeries are replaced by new modalities of conservative treatment, which involve decompression and marsupialization in specific cases [4]. This aspect is addressed by the use of decompression, during which a small hole is created in the cystic cavity and kept patent by insertion of a drainage tube or self-designed stent that leads to a reduction in pressure within the cavity, thus causing a gradual decrease in its size [5]. The creation of a surgical window between the cystic cavity and the oral environment by marsupialization, later to be described by Partsch in the late 19th century, followed by suture of the cyst lining to take that of the oral environment [6]. New studies are proving to have good results on such conservative treatments with Karen Fell in a large clinical series; decompression was shown to reduce mean lesion areas by 79.3 percent [7]. In line with this, systematic review studies have strengthened the studies on the benefits of marsupialization in the minimization of cystic lesions, especially with pediatric patients and those with vital anatomical structures [8]. Modern studies have also explained that the age of the patient, size of the cyst and histopathological diagnosis play a role in the effectiveness of the treatment, where younger patients exhibit more positive changes to conservative treatment [9]. Keratocystic odontogenic tumor is highly recurrent and vicious, but when treated with a conservative measure accompanied by adjunctive therapy, it has proved helpful [10]. The recurrence rate was as low as 1.6% in recent studies, where, in the case of decompression enucleation with chemical cauterization is followed [11]. In addition, recent developments in three-dimensional imaging have made it possible to perform accurate volumetric evaluation of cystic reduction, which allows objective investigation of the effectiveness of treatment [12]. Even though there is increasing evidence favouring conservative management, there are still major gaps in our mutual understanding of ideal selection criteria of treatments and long-term outcomes [13]. Some limited comparative studies have been done on the relative effectiveness of decompression and marsupialization and the standard protocols of selection of patients and monitoring of treatment is yet to be defined [14]. Moreover, the effect of the type of cyst, anatomical location and patient demographics on the outcome of treatment is an issue that still needs to be clarified [15]. Therefore, it is of interest to evaluate the clinical effectiveness of cystic decompression and marsupialization procedures in managing odontogenic cysts, comparing treatment outcomes, complications and long-term success rates to establish evidence-based guidelines for conservative cyst management.

## Materials and Methods:

This was a retrospective cohort study that was carried out in the Department of Oral and Maxillofacial Surgery after seeking institutional ethical clearance. Eligibility criteria involved 124 patients with the treatment of odontogenic cysts (January 2018-December 2022) and their age was 12 to 65 years, with radiologically proven cysts, additionally, at least 12 months of follow-up and the presence of full clinical data. The patients with syndromic diseases, previous intervention on the same lesion, metabolic bone diseases, or an inadequate radiographic record were excluded. Decompression (n=68) or marsupialization (n=56) were allocated depending on the closeness of the lesion to vital tissue as well as accessibility to the surgeon. Diagnostic imaging was performed using panoramic radiographs (Orthophos XG Plus, Sirona) and CBCT scans (NewTom VGi evo, QR Systems) in the case of necessity. Small-sized bony windows and self-shaped thermoplastic stents or polyethylene tubes were used for decompression. Marsupialization was done through bigger surgical incisions and the cyst was lined and sutured to the oral mucosa and iodoform gauze was used to pack up the wound. Both groups were subjected to standard post-operative management and an irrigation regime. Every procedure was performed according to the postulates of the Declaration of Helsinki and the internal rules of surgery.

The data gathered in the clinical setting involved demographics, type of cysts and their loci, types of treatment and follow-up results. Cyst size radiographically was measured with calibrated digital software and volumetric reduction was calculated based on a formula of an ellipsoid. The main results included the percentage reduction in cyst size, time used to treat a patient and radiographic increase in the density of the bone. Secondary outcomes were complications, patient satisfaction (20-point) and recurrence. Descriptive statistics were performed in SPSS 28.0 (IBM Corp.) with a p < 0.05 level of significance.

## Results:

## Patient demographics and cyst characteristics:

The study included 124 patients (80 males, 44 females) with a mean age of 34.2 ± 12.8 years. No significant demographic differences were found between the decompression and marsupialization groups (p = 0.294). Radicular cysts were the most common pathology (41.9%), followed by dentigerous cysts (30.6%) and keratocystic odontogenic tumors (18.5%). The mandible was more frequently involved (62.9%) than the maxilla (37.1%) (Table 1 - see PDF).

## Treatment outcomes and subgroup analysis:

Decompression showed significantly greater cyst size reduction (78.4 ± 9.2%) compared to marsupialization (71.6 ± 8.7%, p < 0.001), with a shorter mean treatment duration (8.3 ± 2.1 vs. 11.7 ± 3.4 months, p < 0.001). Radiographic bone density improved in over 83% of cases in both groups without significant difference (p = 0.312). Subgroup analysis showed radicular and dentigerous cysts responded better than keratocystic odontogenic tumors, with decompression consistently outperforming marsupialization (Table 2 - see PDF). Patients ≤25 years had significantly better outcomes with both treatments compared to older patients (Table 3 - see PDF).

## Complications, recurrence and satisfaction:

Complication rates were low and comparable between groups (7.4% for decompression, 12.5% for marsupialization; p = 0.331). Recurrence occurred in 5 cases (4.0%), all involving keratocystic odontogenic tumors, with no significant difference between groups (p = 0.515). Patient satisfaction was high in both groups (mean scores: 8.7 for decompression vs. 8.4 for marsupialization, p = 0.189) and most patients reported minimal treatment-related discomfort (Table 4 - see PDF).

The current retrospective study illustrates that both decompression and marsupialization have apparent clinical efficacy in the treatment of odontogenic cysts, but the former is more effective in regard to reduction of cyst volume and healing time. The results are consistent with the recent systematic reviews pointing out the effectiveness of conservative strategies in the management of cystic lesions [16]. The mean reduction rate (78.4%) in decompression we have observed closely compares with the one reported in a large multi-center study (79.3%) [17], which conclusively proves that the results can be reproduced in other settings of clinical practice. The better results of decompression as compared to marsupialization in our research lead to the outcome to be against some experiences in the past that showed similar merit in both forms of treatment [18]. Such discrepancies can be explained by variations in patient selection criteria, cystic characteristics and guidelines on treatment. The predictable method of decompression with custom-made stents might have fostered better results because they provided similar drainage and, thus, overcame early closure of the decompression point [19]. The described age-dependent response to treatment used in our cohort also supports the existing literature that shows an increased healing potential in younger patients [20]. The significantly improved results in patients, whereas there is no interest in loss of developing dentition and growth centers [21]. This age-related reaction is probably due to the higher metabolism and the ability to regenerate younger bone tissue [22]. Analysis within each of the cyst types showed varying responses to treatment, with radicular cysts showing the best results. This fact corroborates the inflammatory character of radicular cysts that might be more susceptible to the pressure decrease and drainage [23]. The less predictable course witnessed in keratocystic odontogenic tumors explains the fact that they exhibit aggressive biological behavior and an innate propensity to recurrence [24]. The fact that only recurrence happened in patients with keratocystic odontogenic tumors makes it important to have longer follow-up and other possible extra-treatment options when dealing with such lesions [25].

The safety of conservative modalities as opposed to radical surgery intervention is corroborated by the relatively low rates of complications in the two treatment groups [26]. The lack of significant adverse events in the form of pathological fractures or everlasting neurosensory losses is favorable as compared to the literature on the complication rate of enucleation procedures [27]. Our findings of transient paresthesia in the series are similar to other literature studies of recovery of neural functioning with conservative management of cyst [28]. The results show the essence of the choice of patients and method standardization in obtaining the best results. Such favorable results likely occur due to the utilization of custom-made decompression devices and universal irrigation plans [29]. The use of three-dimensional imaging in volumetric measurement yields objective data relating to the effectiveness of treatment and limitations of the two-dimensional radiographic analysis [30]. Among the strong aspects of the study, one can name the fact that the sample size was rather big, the protocols of treatment were standardized and the assessment of the outcome was complete. In the long term, the follow-up period gives a meaningful assessment of the stability of the long-term treatment and the frequency of recidivism. Nevertheless, a number of limitations have to be noted. There is a possibility that the retrospective design carried with it a possibility of selection bias, as well as the fact that the treatment allocation was not randomized, leading to a possible influence on the outcomes. Lack of unified points of comparison on the choice of technique might have influenced the comparison of the treatment groups. Relatively greater efficiency of decompression as opposed to marsupialization will have to be determined conclusively by future prospective studies that are randomized and controlled. Further, research on additional modalities that may complement bone substitutes or platelet-rich fibrin will also improve the effectiveness of conservative cyst management [31]. Optimal treatment choice, based on clinical decision-making, may be enhanced by developing predictive models that will consider patient and cyst characteristics [32]. Clinical implications of our results are additional indications of using conservative methods of dealing with odontogenic cysts, especially in large cysts and applying to young individuals. A combination of the demonstrated efficacy and safety profile supports the idea to consider these techniques as the first-line treatments and apply radical surgical intervention only in cases with definite indications or failure of other methods [33].

## Conclusion:

This retrospective study establishes that decompression and marsupialization are effective conservative modalities of treating odontogenic cysts with similarities in cases that require them. In younger patients, decompression offered better results in size reduction and duration of treatment. The results showed support for the usage of these low-morbidity methods in regular clinical practice as an alternative method to enucleation.
